# Allogeneic fibroblasts vs conventional debridement after successful endovascular interventions for treating chronic ulcers induced by peripheral artery disease

**DOI:** 10.20452/wiitm.2025.17959

**Published:** 2025-07-04

**Authors:** Azat Chinaliyev, Azat Chinaliyev, Bazylbek Zhakiyev, Didar Khassenov, Gulnara Sakhipova, Natalya Zagorulya, Gaukhar Kuanyshbayeva, Nurlan Zhampeissov, Damir Biktashev, Murat Jakanov, Ainur Donayeva, Ibrahim A. Abdelazim

**Affiliations:** Department of Surgical Diseases No. 2, West Kazakhstan Marat Ospanov Medical University, Aktobe, Kazakhstan; Department of Interventional Radiology, National Research Oncology Center, Astana, Kazakhstan; Department of Science and Education, West Kazakhstan Marat Ospanov Medical University, Aktobe, Kazakhstan; Department of General Medicine, West Kazakhstan Marat Ospanov Medical University, Aktobe, Kazakhstan; Department of Internal Diseases and Geriatrics, Astana Medical University, Astana, Kazakhstan; Department of Fundamentals of Medicine, Astana Medical University, Astana, Kazakhstan; Department of Radiation Diagnostics and Ultrasound Imaging, National Scientific Medical Centre, Astana, Kazakhstan; Department of Internal Diseases, Gastroenterology, Endocrinology and Pulmonology, Astana Medical University, Astana, Kazakhstan; Department of General Surgery, West Kazakhstan Marat Ospanov Medical University, Aktobe, Kazakhstan; Department of Normal Physiology, West Kazakhstan Marat Ospanov Medical University, Aktobe, Kazakhstan; Department of Obstetrics and Gynecology, Faculty of Medicine, Ain Shams University, Cairo, Kazakhstan

**Keywords:** allogeneic fibroblasts, chronic ulcers, conventional debridement, endovascular interventions, peripheral artery disease

## Abstract

**INTRODUCTION:**

Fibroblasts are stromal and connective tissue cells that play crucial roles in the intracellular matrix and granulation tissue synthesis during tissue proliferation. They are also responsible for epithelialization and healing of skin lesions.

**AIM:**

Our aim was to compare the use of allogeneic fibroblasts with conventional debridement after successful endovascular interventions (EVIs) for the treatment of chronic ulcers induced by peripheral artery disease (PAD).

**MATERIALS AND METHODS:**

A total of 116 participants with chronic ulcers due to PAD were randomly assigned, after successful EVI, to receive either allogeneic fibroblasts (study group; n = 58) or conventional debridement (control group; n = 58) for treatment of the ulcers. The participant data were collected over 1 year of follow-up to compare the effectiveness of both methods.

**RESULTS:**

The mean (SD) duration of initial and complete healing of chronic ulcers after successful EVI was shorter in the allogeneic fibroblast group (2.59 [0.53] and 5.04 [0.58] months, respectively) than in the controls (3.56 [0.44] and 5.8 [0.35] months, respectively; *P* <⁠0.001 for all). A correlation analysis showed a moderately significant correlation between the healing of chronic ulcers (both initial and complete) after successful EVI and the use of allogeneic fibroblasts.

**CONCLUSIONS:**

Allogeneic fibroblasts are an effective and noninvasive option for the treatment of chronic PAD-induced ulcers after successful EVI. The duration of initial and complete healing was significantly shorter in the allogeneic fibroblast group than in the conventional debridement group.

## INTRODUCTION

Peripheral artery disease (PAD) is a chronic occlusive vascular disorder affecting the lower limb arteries.^1^ Worldwide, approximately 202 million adults suffer from PAD.^2^ The risk of PAD increases with age, smoking, diabetes mellitus, and hypertension.^3^ PAD may present with intermittent claudication and / or chronic ulcers or can be asymptomatic.^1^ About 70% of chronic ulcers due to PAD recur after treatment.^4^ The ulcers affect the quality of life (QoL) of more than 40 million individuals worldwide.^5^ In the United States, the estimated annual cost of their treatment is approximately 15 billion USD.^5^

**TABLE 1:** Rutherford’s classification for lower limb ischemia

Grade	Category	Clinical symptoms	Classification criteria
0	0	Asymptomatic No significant occlusive disease	Complete treadmill exercise
	1	Mild claudication	Ankle pressure >50 mm Hg after treadmill exercise but at least 20 mm Hg lower than the resting value
I	2	Moderate claudication	Between categories 1 and 3
	3	Severe claudication	Cannot complete treadmill exercise and ankle pressure after exercise <50 mm Hg
II	4	Ischemic rest pain	Resting ankle pressure <40 mm Hg and flat or barely pulsatile ankle
III	5	Minor tissue loss (nonhealing ulcers with focal gangrene)	Resting ankle pressure <60 mm Hg, flat or barely pulsatile ankle, and toe pressure <40 mm Hg
	6	Major tissue loss (loss extending above transmetatarsal level and nonsalvageable functional foot)	Same as category 5

Category 0–3: noncritical lower limb ischemia

Category 4–6: critical lower limb ischemia

**TABLE 2:** Wound, ischemia, and foot infection classification of the Society for Vascular Surgery Lower Extremity Guidelines Committee

Class	Symptoms and management
0	No ulcer and no gangrene. Clinical symptoms: ischemic pain at rest (typical symptoms + grade 3 ischemia) and no ulcer.
I	Small shallow ulcers on the distal leg or foot, absence of exposed bone unless limited to the distal phalanx, and no gangrene. Clinical symptoms: slight loss of tissue (wound defect). Can be saved with amputation (1 or 2 toes) or skin covering.
II	Deeper ulcer with exposed bone, joint or tendon, usually without a heel ulcer or shallow heel ulcer without calcaneus involvement and gangrenous changes limited to the toes. Clinical symptoms: major tissue loss that can be salvaged with multiple (≥3) digital amputations or standard TMA and/or skin coverage.
III	Extensive deep ulcer on the forefoot and/or midfoot; deep full-thickness heel ulcer and/or calcaneus involvement and extensive gangrene involving the forefoot and/or midfoot (full thickness heel necrosis and/or calcaneus involvement). Clinical symptoms: extensive tissue loss can only be salvaged with complex foot reconstruction or unconventional TMA, flap coverage or complex wound management for large soft tissue defects.

Abbreviations: TMA, transmetatarsal amputation

The treatment of chronic ulcers due to PAD is impossible without improving blood flow through the ischemic area. Endovascular interventions (EVIs) are minimally-invasive options for treating PAD.^6^ Fibroblasts are stromal and connective tissue cells that play crucial roles in the intracellular matrix and granulation tissue synthesis during tissue proliferation.^7^ They are responsible for epithelialization, healing of skin lesions, renewal of the epidermis, activation of keratinocytes, and synthesis of collagen and elastin.^8^ Several studies have reported the crucial role of fibroblasts in the healing process.^9^**^,^**^10^

Based on the pathogenesis of chronic ulcers and the roles of fibroblasts in epithelialization and healing, this study hypothesized that EVIs can improve blood flow through the ischemic area and that allogeneic fibroblasts could aid in the healing of PAD-induced chronic ulcers.

## AIM

This study aimed to compare the use of allogeneic fibroblasts with conventional debridement after successful EVIs for the treatment of chronic ulcers due to PAD.

## MATERIALS AND METHODS

The study was carried out between January 2023 and September 2024 at the National Research Oncology Center (Astana, Kazakhstan), and included 116 participants with chronic PAD-induced ulcers randomly assigned to receive either allogeneic fibroblasts (study group) or conventional debridement (controls).

EVIs were conducted at the interventional radiology unit of the National Research Oncology Center (Astana, Kazakhstan). The surgical unit of the same institution was the venue of patient admission and the use of allogeneic fibroblasts or conventional debridement.

The participants’ age ranged from 18 to 75 years. They all suffered from chronic ulcers due to PAD and failed medical treatment (eg, failed glycemic and blood pressure control and ineffective antiplatelets and anticoagulants), and underwent successful EVIs for PAD.

The diagnosis of PAD in this study was based on ankle-brachial index (ABI) below 0.8^11^ and the Rutherford’s classification (Table 1),^12^ and confirmed on duplex ultrasound and computed tomography angiography (CTA).^13^ The ABI is the ratio of the ankle’s systolic blood pressure to the arm’s systolic blood pressure.^11^**^,^**^14^ Chronic ulcers were defined as ulcers that showed no signs of healing after 3 months of appropriate treatment or were not completely healed after 12 months of appropriate treatment.^15^ Successful EVIs for PAD indicated that the targeted arterial portions (TAPs) were recanalized or their stenosis after EVI was smaller than 30%.^16^

Participants with unsuccessful EVI, infected chronic ulcers, congestive heart failure (New York Heart Association class III or IV),^17^ prior cardiac surgery, acute myocardial infarction, acute cerebrovascular accident, history of embolic disorders, impaired renal function, or allergy to radio-opaque dye, or who were pregnant, breastfeeding, or declined to participate were excluded from the study.

**TABLE 3:** Grades of chronic ulcer infection

Class	Symptoms and management
0	No symptoms or signs of infection.
I	Local infection involving only the skin and subcutaneous tissue (without involvement of deeper tissues and without systemic signs). Local infection is defined as the presence of at least 2 of the following: 1) local pain and swelling; 2) induration or erythema >0.5 to ≤2 cm around the ulcer; 3) purulent discharge
II	Local infection involving structures deeper than the skin and subcutaneous tissues (eg, abscess formation, osteomyelitis, septic arthritis, fasciitis) and no evidence of a systemic inflammatory response.<br>Local infection is defined as the presence of at least 2 of the following:<br>1) local pain and swelling; 2) induration or erythema >2 cm; 3) purulent discharge
III	Local infection with features of systemic inflammatory response, manifested with at least 2 of the following: 1) temperature >38°C or <36°C; 2) heart rate >90 bpm; 3) respiratory rate >20/min; 4) TLC >12 000 cells/mm³

Abbreviations: TLC, total leukocyte count

Unsuccessful EVIs were defined as TAPs that remained occluded or showed over 30% stenosis after the procedure.^16^

The study participants were treated in accordance with the Republic of Kazakhstan management protocol for lower limb angioplasty after the West Kazakhstan Medical University ethical committee approval (No 10.5; protocol No 10). All patients gave their written informed consent. This study was registered as a clinical trial (NCT06724276) and registered within the the Republic of Kazakhstan Ministry of Health (No 207/2020) and the Republic of Kazakhstan Ministry of Justice (No 21683).

Prior to EVIs, the participants were hospitalized for blood sugar and blood pressure monitoring and to discontinue antiplatelets and anticoagulants. Routine investigations were conducted after the hospitalization, including electrocardiograms and renal, liver, and coagulation profiles, as well as hepatitis and HIV screening. Radiological evaluations including chest radiology, abdominal ultrasound, arterial duplex ultrasound, and CTA were also performed.

The ischemic area was carefully assessed using the Wound, Ischemia, and Foot Infection (WIfI) classification to identify changes in the skin and muscles, and to evaluate chronic ulcers^18^ (TABLE 2).

The participants with chronic ulcers were also assessed for infection, and those with infected ulcers were excluded from the study (infection disrupts healing of chronic ulcers and worsens tissue damage^19^; TABLE 3).

EVIs were performed using the retrograde technique to access the common femoral artery under aseptic conditions and local anesthesia.^20^ The sites of vascular occlusion (TAPs) were identified through imaging and instillation of a radio-opaque dye.

In this study, EVIs were aimed at re-establishing blood flow through 1 TAP in each EVI session.^11^ Two vascular surgeons and 2 interventional radiologists attended each EVI session to determine the appropriate EVI (eg, either balloon angioplasty or vascular stenting) for each participant based on their experience and the TAP diameter.

Percutaneous transluminal angioplasty (PTA) was performed using a Passeo-35 balloon (Biotronic, Berlin, Germany), and a CGuard embolic prevention stent (InspireMD, Brussels, Belgium) was used for vascular stenting if a TAP did not respond to PTA.

Post-EVI angiography was conducted in all participants to assess the result of each EVI on the TAP, followed by application of a pressure bandage for the femoral puncture site and 24 hours of bed rest.

After EVIs, proper glycemic and blood pressure control was ensured, and antiplatelet and anticoagulant therapies were resumed when indicated.

Following successful recanalization, the participants were randomized using computer-based randomization to receive either allogeneic fibroblasts (study group; n = 58) or conventional debridement (control group; n = 58) for the treatment of chronic ulcers.

Allogeneic fibroblasts are a noninvasive treatment for chronic ulcers.^21^ The fibroblasts used in this study were extracted from neonatal foreskin under aseptic conditions, subjected to biological and serological evaluation to ensure their safety for human use, and then cultured at the Astana National Center of Biotechnology. The cultured fibroblasts were mixed in a 10-ml spray dispenser (2 million fibroblasts per 1 ml of the spray) and stored in vials at –86 ºC.

After thawing for 5 minutes, the caps of the vials were replaced with pump heads. The vials were gently inverted to resuspend the fibroblasts before use. Allogeneic fibroblasts were sprayed once daily to completely cover the chronic ulcers.

The chronic ulcers in the control group were considered healable after successful EVIs. They were managed using surgical (conventional) debridement (the gold standard) to enhance granulation and create a moist environment to promote healing.^22^

Surgical debridement was performed twice a week in an operating theater (ie, sterile environment) with adequate pain control.^23^

Participant data, including age, sex, smoking status, body mass index (BMI, kg/m^2^), medical disorders, the Rutherford’s and WIfI classifications, EVIs, and chronic ulcer healing (initial and complete), were collected over a 1-year follow-up period from the participants during clinical evaluation and from their medical records.

The initial signs of healing in chronic ulcers included re-epithelialization and the formation of granulation tissue (mediated by fibroblasts and endothelial cells), followed by a reduction in the size of the ulcers and their physical contraction (mediated by myofibroblasts).^24^

**TABLE 4:** Characteristics of the study patients

Variable			Allogeneic fibroblast group (n = 58)	Conventional debridement group (n = 58)	95% CI	P value
Age, y, mean (SD)			55.7 (5.04)	57.3 (5.04)	–3.29 to 0.09	0.06
Sex, n (%)	Men		43 (74.1)	37 (63.8)	–	0.6
	Women		15 (25.9)	21 (36.2)		
BMI, kg/m², mean (SD)			27.4 (2.1)	27.7 (1.97)	–1.05 to 0.45	0.4
Smoking, n (%)			28 (48.3)	24 (41.4)	–	0.6
Medical disorders, n (%)						
Diabetes			24 (41.4)	36 (62.1)	–	0.2
Hypertension			25 (43.1)	22 (37.9)	–	0.7
Renal diseases			11 (19)	8 (13.8)	–	0.5
Cardiac disorders			6 (10.3)	3 (5.2)	–	0.3
Hypertension + diabetes			8 (13.8)	4 (6.9)	–	0.3
Multiple disorders			4 (6.9)	3 (5.2)	–	0.7
Rutherford's classification, n (%)						
Noncritical lower limb ischemia		Grade I, Category 2	23 (39.7)	29 (50)	–	0.5
		Grade I, Category 3	12 (20.7)	14 (24.2)		
Critical lower limb ischemia		Grade II, Category 4	10 (17.2)	6 (10.3)		
		Grade III, Category 5	13 (22.4)	9 (15.5)		
WIfI classification of chronic ulcers, n (%)						
Class I			38 (65.5)	42 (72.4)	–	0.7
Class II			15 (25.9)	14 (24.2)		
Class III			5 (8.6)	2 (3.4)		
TAP, n (%)						
External iliac			6 (10.3)	9 (15.5)	–	0.5
Superficial femoral			23 (39.7)	24 (41.4)		
Popliteal			11 (19)	13 (22.4)		
Anterior tibial			8 (13.8)	7 (12.1)		
Posterior tibial			10 (17.2)	5 (8.6)		
EVL, n (%)						
Balloon angioplasty (PTA)			52 (89.7)	49 (84.5)	–	0.8
Vascular stenting			6 (10.3)	9 (15.5)		
Nature of vascular lesion in TAPs, n (%)						
Balloon angioplasty (PTA)		Total	52 (89.7)	49 (84.5)	–	0.66
		Segmental stenosis	47 (52.8)	42 (47.2)		
		Vascular occlusion	5 (41.7)	7 (58.3)		
Vascular stenting		Total	6 (10.3)	9 (15.5)	–	0.63
		Segmental stenosis	1 (33.3)	2 (66.7)		
		Vascular occlusion	5 (41.7)	7 (58.3)		
Chronic ulcer healing, mean (SD)						
Initial healing			2.59 (0.53)	3.56 (0.44)	–1.15 to –0.79	<0.001
Complete healing			5.04 (0.58)	5.8 (0.35)	–0.94 to –0.58	<0.001

Data presented as number and percentage were analyzed with the χ2 test, and those presented as mean (SD), with the *t* test.

Abbreviations: BMI, body mass index; EVI, endovascular intervention; PTA, percutaneous transluminal angioplasty; TAP, targeted arterial portion; WifI, wound, ischemia, and foot infection

### Statistical analysis

A sample size of at least 100 participants in 2 groups was required to produce an acceptable Figure for this study, as calculated using the Epi InfoTM package (StatCalc, version 7.2.5, Atlanta, Georgia, United States) with 2-sided 95% CI, 80% power, 1:1 ratio of the study to control groups, and a 0.5 risk coefficient. The statistical analysis was conducted using the χ^2^ test and the *t *test. The Pearson correlation was used to determine the relationship between chronic ulcer healing after EVIs and the use of allogeneic fibroblasts or conventional debridement. A *P* value below 0.05 was considered significant.

## RESULTS

A total of 116 participants with chronic ulcers due to PAD and after successful EVIs were included in this comparative study. The participants were randomly assigned into the study group (n = 58) receiving allogeneic fibroblasts or the control group (n = 58) treated with conventional debridement for their chronic ulcers.

**FIGURE 1:**
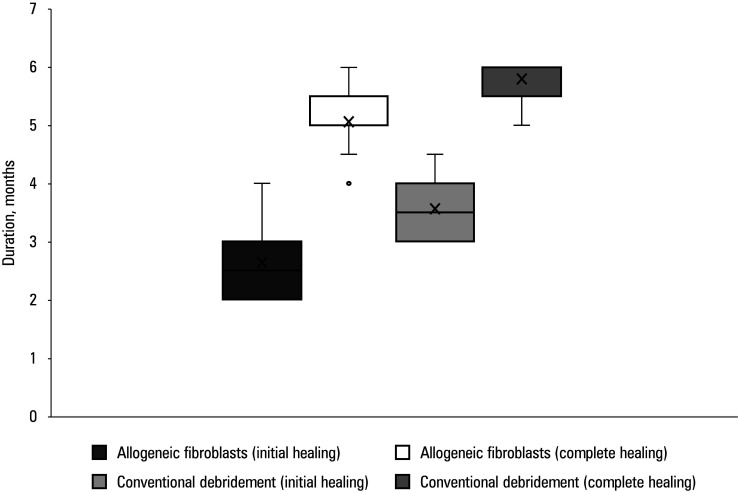
Initial and complete healing of chronic ulcers after endovascular interventions; whiskers indicate the minimal and maximal values, boxes indicate the interquartile range, X represents the mean value, and the central line represents one value lower than the minimal value for allogeneic fibroblast complete healing.

No substantial differences were reported between the study and control groups regarding mean (SD) age (55.7 [4.12] vs 57.3 [5.04] y; *P* = 0.06) and BMI (27.4 [2.1] vs 27.7 [1.97] kg/m^2^; *P* = 0.4).

Similarly, no marked differences were found between the allogeneic fibroblast group and the controls regarding the percentage of men (74.1% vs 63.8%; *P* = 0.6), smokers (48.3% vs 41.4%; *P* = 0.6), individuals with diabetes (41.4% vs 62.1%; *P* = 0.2), hypertension (43.1% vs 37.9%; *P* = 0.7), renal diseases (19% vs 13.8%; *P* = 0.5), cardiac disorders (10.3% vs 5.2%; *P* = 0.3), or multiple medical disorders (6.9% vs 5.2%; *P* = 0.7; TABLE 4).

### Targeted arterial portions and endovascular interventions

The external iliac artery was the TAP in 6 patients (10.3%) from the allogeneic fibroblast group and 9 controls (15.5%). The superficial femoral artery was the TAP in 23 cases (39.7%) in the allogeneic fibroblast group and 24 controls (41.4%). The popliteal artery was the TAP in 11 participants (19%) from the allogeneic fibroblast group and 13 controls (22.4%). The anterior tibial artery was the TAP in 8 cases (13.8%) in the allogeneic fibroblast group and 7 controls (12.1%). The posterior tibial artery was the TAP in 10 patients (17.2%) from the allogeneic fibroblast group and 5 patients (8.6%) in the control group (*P* = 0.5 for all).

PTA was used to manage TAPs in 52 participants (89.7%) from the allogeneic fibroblast group and 49 controls (84.5%). Vascular stenting was used in 6 cases (10.3%) in the allogeneic fibroblast group and 9 controls (15.5%; *P* = 0.8; TABLE 4).

PTA was used to manage TAPs with segmental stenosis in 89 patients (88.1%), including 47 individuals (52.8%) from the allogeneic fibroblast group and 42 controls (47.2%), and to manage vascular occlusions in 12 patients (11.9%), including 5 (41.7%) in the allogeneic fibroblast group and 7 controls (58.3%) (*P* = 0.66). Vascular stenting was used to manage TAPs with vascular occlusions in 12 cases (80%), including 5 (41.7%) in the allogeneic fibroblast group and 7 (58.3%) in the control group, and to manage segmental stenosis in 3 participants (20%), including 1 patient (33.3%) in allogeneic fibroblast group and 2 controls (66.7%; *P* = 0.63; TABLE 4).

### Chronic ulcer healing

The mean (SD) duration of the initial and complete healing of chronic ulcers after successful EVIs was shorter in the study group (2.59 [0.53] and 5.04 [0.58] mo) than in the controls (3.56 [0.44] and 5.8 [0.35] mo; *P* <⁠0.01 for all; TABLE 4 and FIGURE 1).

**FIGURE 2:**
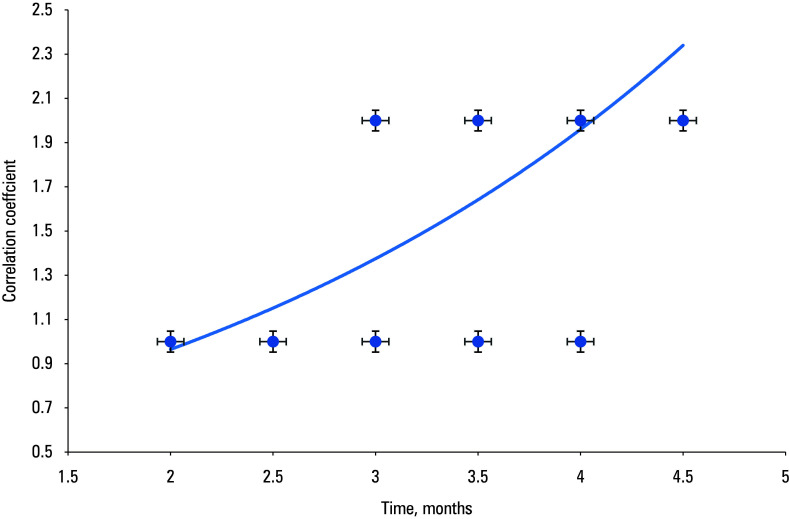
Correlation between initial healing of chronic ulcers after endovascular interventions and the use of allogeneic fibroblasts (A) vs conventional debridement (B)

**FIGURE 3:**
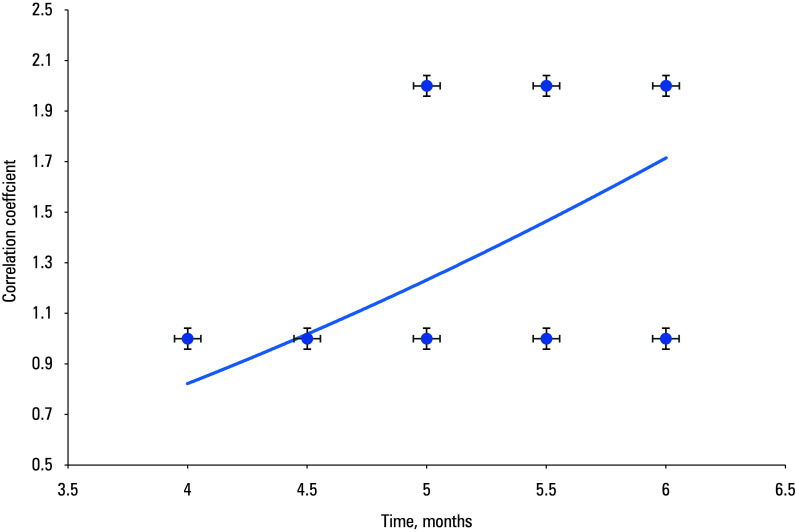
Correlation between complete healing of chronic ulcers after endovascular interventions and the use of allogeneic fibroblasts (A) vs conventional debridement (B)

The correlation analysis showed a moderately significant correlation between the healing of chronic ulcers (both initial and complete) after successful EVIs and the use of allogeneic fibroblasts. The healing times were shorter in the study group than in the controls both for the initial (r = 0.7065; *P* <⁠0.001; FIGURE 2) and complete healing (r = 0.6208; *P* <⁠0.001; FIGURE 3).

## DISCUSSION

Chronic PAD-induced ulcers affect the QoL of more than 40 million individuals worldwide.^5^ In the United States, the estimated annual cost of treating such ulcers is approximately 15 billion USD.^5^

The treatment of chronic ulcers is impossible without improving blood flow through the ischemic area. EVIs are a minimally-invasive option for treating PAD.^6^

Fibroblasts are stromal and connective tissue cells that play a crucial role in the intracellular matrix and granulation tissue synthesis during tissue proliferation.^7^ They are responsible for epithelialization, healing of skin lesions, and renewal of the epidermis.^8^

Based on the pathogenesis of chronic ulcers and the roles of fibroblasts in epithelialization and healing, this study hypothesized that EVIs can improve blood flow through the ischemic area and that allogeneic fibroblasts could aid in the healing of chronic ulcers due to PAD.

Therefore, 116 participants with PAD-induced chronic ulcers and successful EVIs were included in this study comparing allogeneic fibroblasts with conventional debridement after EVIs for treating the ulcers.

The diagnosis of PAD in this study was based on an ABI below 0.8^11^ and the Rutherford’s classification,^12^ and it was confirmed by duplex ultrasound and CTA.^13^

The study participants were treated in accordance with the Republic of Kazakhstan management protocol for lower limb angioplasty. Post-EVI angiography was conducted in all participants to assess the results of each EVI on the TAP. Successful EVIs for PAD indicated that the TAPs were recanalized or had below 30% stenosis.^16^

After successful EVIs, the participants were randomized using computer-based randomization to receive either allogeneic fibroblasts (study group; n = 58) or conventional debridement (control group; n = 58) for the treatment of chronic ulcers.

The participant data were collected over a 1-year follow-up period to compare allogeneic fibroblasts with conventional debridement after EVIs for treating PAD-related chronic ulcers.

No significant differences were reported between the allogeneic fibroblast group (study group) and the conventional debridement group (controls) in terms of mean age and BMI.

Similarly, no significant differences were found between the allogeneic fibroblast group and the controls regarding the percentage of men, women, smokers, individuals with diabetes, hypertension, renal diseases or cardiac disorders, and participants with multiple medical disorders.

The patients from the allogeneic fibroblast group and controls were also statistically matched, with no difference in the number of participants included from each Rutherford’s classification category or from each WIfI classification class.

### Targeted arterial portions and endovascular interventions

PTA was used to manage TAPs in 89.7% of the participants in the allogeneic fibroblast group and 84.5% of the controls. Vascular stenting was used to manage TAPs in 10.3% of the study group patients and 15.5% of the controls.

PTA was used to manage TAPs with segmental stenosis in 88.1% of the participants, and to manage vascular occlusions in 11.9% of the participants. Vascular stenting was employed to manage TAPs with vascular occlusions in 80% of the patients, and to manage segmental stenosis in 20% of the participants.

PTA involves intravascular inflation of a balloon to compress atheroma against the TAP wall.^25^

PTA is indicated for short-segment stenosis, while vascular stenting should be preferred in long and complex femoropopliteal lesions.^26^ The 1-year patency rate is much lower after PTA in long vascular lesions (>10 cm).^27^

In a prospective study, no major amputations were reported, with 100% limb salvage at 15 months after PTA for complicated popliteal PAD.^28^

A randomized study^29^ showed similar outcomes for PTA and self-expanding stents in the treatment of superficial femoral lesions. The BASIL trial (Bypass Versus Angioplasty in Severe Ischaemia of the Leg)^30^ reported similar amputation-free rates at 12 and 36 months after EVIs. A meta-analysis showed significant rates of limb salvage after PTA for severe tibial artery lesions.^31^ A Cochrane review^32^ did not reach a conclusion on PTA vs primary stenting for iliac lesions due to insufficient evidence.

Previous trials, such as THUNDER (Local Taxan With Short Time Contact for Reduction of Restenosis in Distal Arteries),^33^ LEVANT 2 (Moxy Drug Coated Balloon vs Standard Balloon Angioplasty for the Treatment of Femoropopliteal Arteries),^34^ BIOLUX P-I (A Prospective, Multi-centre, Randomized Controlled, First in Man Study to Assess the Safety and Performance of the Passeo-18 Lux Paclitaxel Releasing PTA Balloon Catheter vs. the Uncoated Passeo 18 Balloon Catheter in Patients With Stenosis and Occlusion of the Femoropopliteal Arteries),^35^ AcoArt-I (Prospective, Multi-center and Randomized Controlled Clinical Study to Verify Effectiveness and Safety of Drug-eluting Balloon in PTA Procedure),^36^ IN.PACT (Randomized Trial of IN.PACT Admiral Drug Coated Balloon vs Standard PTA for the Treatment of SFA and Proximal Popliteal Arterial Disease),^37^ and ILLUMENATE (Pivotal Trial of a Novel Paclitaxel-Coated Percutaneous Angioplasty Balloon)^38^ have reported significant patency rates after using paclitaxel balloons in EVIs.

A paclitaxel balloon, which delivers an antiproliferative drug to TAPs, was approved by the Food and Drug Administration and is considered an innovative EVI for treating femoral, popliteal, and tibial PAD.^39^

### Chronic ulcer healing

The mean (SD) duration of initial and complete healing of chronic ulcers after successful EVIs was significantly shorter in the study group (2.59 [0.53] and 5.04 [0.58] months) than in the controls (3.56 [0.44] and 5.8 [0.35] months; *P* <⁠0.001 for all).

The correlation analysis showed a moderately significant correlation between the healing of chronic ulcers (both initial and complete) after successful EVIs and the use of allogeneic fibroblasts.

The initial healing time of chronic ulcers after EVIs was markedly shorter in the study group than in the controls (r = 0.7065; *P* <⁠0.001), and the same outcome was achieved for complete healing (r = 0.6208; *P* <⁠0.001).

Torrealba et al**^1^**^16^ reported an 85% rate of chronic ulcer healing after EVIs (PTA and coated balloons) for TAPs.

Autologous skin grafts are commonly used to treat leg ulcers.^21^ However, they require creation of a donor site^40^ and have not been shown to be effective.^41^

Cells derived from skin biopsies or plucked hair follicles can be used to fabricate cell sheets with results similar to those of split-thickness skin grafts.^42^ Additionally, allogeneic cells do not initiate an adaptive immune response.^43^

Falanga et al^44^ reported rapid healing of venous ulcers after using a skin substitute from neonatal foreskin containing allogeneic keratinocytes and fibroblasts.

In an animal model, Yoon et al^45^ found that human embryonic fibroblasts (HEFs) showed a significant increase in the expression of collagen (types I and III) and fibronectin, with enhanced vessel formation and wound healing. They concluded that HEFs were a promising cell therapy for the treatment of skin ulcers.^45^

Nagase et al^46^ compared the effects of allogeneic and autologous fibroblast sheets on wound healing in an animal model. They found that allogeneic fibroblasts had wound-healing rates comparable to those of autologous fibroblasts.^46^

In a randomized controlled trial, participants with venous ulcers from 28 centers in the United States and Canada were treated with a spray containing allogeneic fibroblasts and keratinocytes for 12 weeks.^21^ The author of this study concluded that the use of the allogeneic fibroblast and keratinocyte spray (at a dose of 0.5 × 10^6 ^cells/ml) every 14 days could improve chronic venous ulcer healing.

This was the first comparative study conducted according to the Republic of Kazakhstan protocol to compare allogeneic fibroblasts with conventional debridement after successful EVIs for treating PAD-related chronic ulcers.

The limited number of human studies investigating allogeneic fibroblasts for the treatment of chronic ulcers and lack of follow-up for cases with unsuccessful EVIs were the limitations of this study. Further studies are required to confirm the effectiveness of allogeneic fibroblasts for the treatment of chronic ulcers due to PAD.

## CONCLUSIONS

Allogeneic fibroblasts are an effective and noninvasive option after successful EVIs for the treatment of PAD-induced chronic ulcers. The d uration of initial and complete healing of chronic ulcers after successful EVIs was significantly shorter in the allogeneic fibroblast group than in the conventional debridement group. Further studies are required to confirm the effectiveness of allogeneic fibroblasts for the treatment of PAD-related chronic ulcers.

## JOURNAL INFORMATION

Videosurgery and Other Miniinvasive Techniques is an official journal of the Videosurgery Foundation.
